# Reproductive Chances of Men with Azoospermia Due to Spermatogenic Dysfunction

**DOI:** 10.3390/jcm10071400

**Published:** 2021-03-31

**Authors:** Caroline Kang, Nahid Punjani, Peter N. Schlegel

**Affiliations:** Department of Urology, Weill Cornell Medical College, New York, NY 10021, USA; cak4005@med.cornell.edu (C.K.); nap4001@med.cornell.edu (N.P.)

**Keywords:** non-obstructive azoospermia, infertility, intracytoplasmic sperm injection

## Abstract

Non-obstructive azoospermia (NOA), or lack of sperm in the ejaculate due to spermatogenic dysfunction, is the most severe form of infertility. Men with this form of infertility should be evaluated prior to treatment, as there are various underlying etiologies for NOA. While a significant proportion of NOA men have idiopathic spermatogenic dysfunction, known etiologies including genetic disorders, hormonal anomalies, structural abnormalities, chemotherapy or radiation treatment, infection and inflammation may substantively affect the prognosis for successful treatment. Despite the underlying etiology for NOA, most of these infertile men are candidates for surgical sperm retrieval and subsequent use in intracytoplasmic sperm injection (ICSI). In this review, we describe common etiologies of NOA and clinical outcomes following surgical sperm retrieval and ICSI.

## 1. Introduction

Infertility affects up to 15% of couples worldwide, with up to 50% of cases attributable to male factor infertility [1]. In a majority of cases, the precise etiology underlying infertility in the male partner remains unclear. A subset of men with infertility have no sperm in the ejaculate, known as azoospermia, which may further be classified into obstructive (OA) or non-obstructive azoospermia (NOA). The majority of cases of NOA are idiopathic, however some known etiologies include genetic disorders, chemotherapy or radiation, developmental or structural abnormalities, and hormonal imbalances (Table 1). Despite the etiology underlying the spermatogenic dysfunction resulting in NOA, sperm often can be surgically extracted from the testis for use in assisted reproductive technology (ART) with varying success. Intracytoplasmic sperm injection (ICSI) requires only a single spermatozoon for injection into an oocyte, and thus has improved the chances for men with NOA to conceive biological children. In this review, we discuss common etiologies for NOA and the reproductive outcomes for NOA men after surgical sperm retrieval and ICSI.

## 2. Treatment of Non-Obstructive Azoospermia

Effective management of infertility in men with NOA requires testicular sperm retrieval as well as ART in the form of ICSI. Since sperm retrieval involves finding one of the very limited sites of sperm production within a highly dysfunctional testis of a man with NOA, it is not surprising that the approach used for sperm retrieval can substantially affect the chance of obtaining sperm for fertility.

A wide variety of approaches have been used for attempted sperm retrieval including fine needle aspiration of the testis (testicular sperm aspiration; TESA), random biopsies of testicular tissue to identify foci of sperm production (testicular sperm extraction; “conventional” TESE) as well as directed testicular surgical sperm retrieval using a microsurgical approach (microdissection testicular sperm extraction; microTESE or mTESE).

Each of these methods were compared using a meta-analysis of published literature [2]. Although a recent meta-analysis reported no difference in sperm retrieval rates when comparing conventional TESE to microTESE, it is important to note that this analysis did not require comparative studies so the heterogeneous nature of NOA patients treated at different sites invalidated any meaningful comparison of surgical techniques [3]. The superiority of microTESE is not surprising, as the surgery directs sampling of testicular tissue to the largest seminiferous tubules, which are those most likely to contain sperm [4]. From a laboratory perspective, the microTESE approach is ideal, as it limits the amount of tissue that must be examined by the andrologist to identify sperm to that which is richest in sperm production. Typical search times to find sperm in isolated, dispersed testicular tissue specimens is only 3 to 5 min at experienced centers. Of note, microTESE, although an invasive surgical procedure, has less effect on testicular function than other approaches for sperm retrieval [4].

## 3. Causes of Non-Obstructive Azoospermia

NOA occurs secondary to the disruption of spermatogenesis within the testicular parenchyma. This disruption of sperm production is a common phenotype with various underlying etiologies. Although understanding the underlying etiology of azoospermia may help in prognosis and counseling, the precise mechanisms by which spermatogenesis is disrupted in these disorders are not well understood. Men with NOA have varying ranges of spermatogenic failure, and even in 30–60% of those with severely dysfunctional histology (i.e., Sertoli Cell Only (SCO) or maturation arrest) small foci of spermatogenesis can be observed [5]. Furthermore, many other presumed idiopathic cases of NOA are likely to be caused by genetic abnormalities that are yet to be fully delineated.

### 3.1. Hormonal Imbalances

Men with hypogonadotropic hypogonadism (HH) suffer from a lack of gonadotropin stimulation, resulting in failure of the testis to produce testosterone or sperm. The defect can be congenital (e.g., Kallmann syndrome, Prader-Willi syndrome) or acquired (e.g., secondary to pituitary tumor or exogenous steroid administration). The resultant phenotype of these men is lack of development of secondary sexual characteristics (with prepubertal phenotype) and infertility. Importantly, because the phenotype is caused by a lack of gonadotropin, treatment of these men with exogenous gonadotropins (e.g., human chorionic gonadotropin (hCG) and recombinant follicle-stimulating hormone (FSH)) can result in the appropriate development of secondary sexual characteristics (i.e., pubic hair development, testis growth, development of muscle mass) and sperm [6]. Men with HH typically do not require ICSI to achieve pregnancy as treatment with exogenous gonadotropins is highly effective in inducing spermatogenesis adequate to allow return of sperm to the ejaculate, which is associated with an increase in endogenous testosterone production [6]. 

Hyperprolactinemia, or elevated serum prolactin levels, is a rare etiology for azoospermia but clinically relevant. Prolactin is produced by the posterior pituitary and elevated levels can result from a prolactin-secreting adenoma (or prolactinoma) [7]. One study examining prolactin levels in infertile men observed increased prolactin levels in men with asthenozoospermia, oligozoospermia, and azoospermia [8]. Since hyperprolactinemia is typically effectively treated with medical therapy, it is rarely a cause for persistent NOA requiring surgical intervention.

### 3.2. Klinefelter Syndrome

Klinefelter Syndrome (KS) is the most common sex chromosome aneuploidy in infertile men, with an estimated prevalence of approximately 10% in men with NOA [9]. This syndrome involves the addition of one or more extra X-chromosome(s), resulting most commonly in a 47,XXY karyotype [9,10,11]. KS is thought to occur secondary to chromosomal nondisjunction during meiosis [12,13]. Physical examination of KS men often reveals characteristic findings of tall stature, reduced testis size, reduced chest and facial hair, gynecomastia, eunuchoid appearance, wide hips, and narrow shoulders [13]. Small testis size is thought to occur due to fibrosis and hyalinization of the seminiferous tubules and is progressive through puberty and adult development [14,15]. Rare, small foci of spermatogenesis in the testes of KS men is hypothesized to be present due to the capability of XXY stem cells to undergo spermatogenesis, or more likely, mitotic errors within the XXY stem cell population resulting in diploid cells capable of completing the remaining spermatogenic process [16]. 

### 3.3. Y-Chromosome Microdeletions

One of the most common identifiable etiologies of NOA are microdeletions of the azoospermia factor (AZF) region of the Y chromosome, and up to 12% of men with NOA harbor AZF microdeletions [17]. There are three loci in the AZF region, which are designated AZFa, AZFb and AZFc, and each locus contains various genes responsible for different aspects of spermatogenesis [18,19,20]. Microdeletions within AZFc are the most common (up to 80%), whereas AZFa (up to 4%) and AZFb (up to 5%) are less common [21]. Polymerase chain reaction (PCR) is used to detect Y-chromosome microdeletions (YCMDs) as these chromosomal deletions are too small to detect by standard karyotype analysis. Current guidelines recommend testing for YCMD along with karyotype analysis in men with NOA or severe oligozoospermia (<5 million sperm/mL) [22]. Knowing the YCMD status in a man with severe infertility carries important prognostic information as the sperm retrieval rates (SRR) in men with complete AZFa and AZFb deletions is zero, whereas men with AZFc deletions can have SRR of approximately 50–60% [23]. 

### 3.4. Malignancy, Chemotherapy, and Radiation

Malignancy and associated treatments such as chemotherapy and radiation are important causes of azoospermia as approximately 50% of men will be affected by cancer in their lifetime [24]. Chemotherapy targets rapidly dividing cells and testicular germ cells which are mitotically and meiotically active and are highly sensitive to these systemic agents [25]. DNA alkylating agents, as well as platinum-containing chemotherapy agents (such as cisplatin), cross-link DNA and are particularly harmful to spermatogonial stem cells and may result in permanent azoospermia [26,27]. Other chemotherapy agents, such as anthracyclines, antimetabolites, and topoisomerase inhibitors, typically are less gonadotoxic and result only in transient decreases in sperm count because differentiating spermatogonia, and not stem cells, are primarily affected [26]. The CED, or cyclophosphamide-equivalent dose, may be used to determine estimated alkylating agent exposure [28]. One study of adult childhood cancer survivors found that when the CED was less than 4000 mg/m^2^ men were normospermic, however there was substantial overlap in the CED values of normozoospermic, oligozoospermic, and azoospermic men [29]. Radiation therapy, on the other hand, may result in irreversible testicular damage secondary to the high radio-sensitivity of testicles [30]. Spermatogonial stem cells are highly sensitive to radiation and are adversely impacted even at low radiation doses (0.1 Gy), with permanent azoospermia typically occurring with doses of 16–20 Gy but reportedly occurring with radiation doses as low as 4 Gy [25,31]. Recovery of spermatogenesis after chemotherapy or radiation therapy depends on the chemotherapy agent used and cumulative chemotherapy or radiation dose [31]. 

Importantly, as the efficacy of cancer treatments has improved, the number of survivors has increased worldwide [24]. Therefore, strong recommendations from the American Urological Association (AUA), American Society of Clinical Oncology (ASCO), and American Society for Reproductive Medicine (ASRM) have been made to counsel and refer patients for discussion of fertility preservation prior to initiating cancer treatments [32,33,34]. Additionally, men undergoing chemotherapy or radiation therapy should avoid pregnancy for a minimum of twelve months after completing treatment because of potentially mutagenic effects of the treatments on germ cells [34,35]. Finally, in men with persistent azoospermia after gonadotoxic chemotherapy or radiation treatment, testicular sperm extraction may be performed to harvest sperm for ART [34].

### 3.5. Cryptorchidism

Cryptorchidism, or undescended testis, is a well-known risk factor for male infertility and a common genitourinary finding. This disorder affects 1–9% of all male neonates worldwide, with approximately 3% of boys remaining cryptorchid at one year of age [36,37]. Semen abnormalities are observed in up to 30% of men with a history of unilateral cryptorchidism and up to 80% of men with a history of bilateral cryptorchidism [38]. Recommended treatment is orchidopexy, or fixation of the testis within the scrotum, and typically is performed early in life (by 18 months of age) to prevent future testicular dysfunction [36,39,40]. The majority of men with a history of cryptorchidism, either unilateral or bilateral, are fertile, however some may develop azoospermia secondary to iatrogenic injury to the testis or testicular vasculature during orchidopexy, or baseline underlying severe spermatogenic dysfunction. For men with NOA secondary to cryptorchidism, testicular sperm extraction is generally favorable with success in the majority of these patients [41,42,43,44,45,46].

### 3.6. Varicocele

Varicoceles are a common cause of infertility, found in at least 5% of men with NOA, and the most common cause of secondary infertility [47,48]. Varicoceles are dilated veins (spermatic and pampiniform plexus veins) within the spermatic cord and may result in subsequent testicular dysfunction [49]. Different mechanisms of testicular dysfunction in men with varicoceles have been proposed, including testicular parenchymal hyperthermia, blood-testis barrier dysfunction, and testicular hypoxia [50]. The ultimate downstream effect is the generation of reactive oxygen species and damage to testicular cells [50,51,52]. Damage to Sertoli, Leydig, and germ cells can result in abnormal or decreased sperm production, as well as deficient testosterone production [49,53]. Although varicoceles clearly contribute to testicular dysfunction, a varicocele is unlikely to be the primary cause of NOA since only 5–10% of men with NOA and clinical varicocele will have enough sperm return to the ejaculate after varicocele repair to avoid testicular sperm extraction [54].

### 3.7. Other Causes of NOA

NOA may also be acquired for a wide variety of reasons including genitourinary infections, such as post-pubertal mumps orchitis, or various classes of medications. Common medication categories with documented negative impacts to fertility include exogenous testosterone or other androgen-modulating medications, psychiatric medications, and anti-hypertensive medications, which all have been documented to modulate the hormonal environment resulting in decreased or absent sperm production [55].

## 4. Optimization of Sperm Production Prior to Surgical Retrieval

Various abnormalities, including hormonal deficiencies and testicular dysfunction, may contribute to abnormal spermatogenesis and decreased or absent sperm production. The production of sperm requires adequate levels of (serum and intratesticular) testosterone, and the goal of optimization is to increase testosterone levels. Serum testosterone levels can be optimized prior to surgical sperm extraction by administration of hormone analogs and modulators, including gonadotropins, aromatase inhibitors (AI), and selective estrogen receptor modulators (SERM) (Table 2). Unfortunately, there is no high-level evidence to support use of medical therapy prior to sperm retrieval, despite many anecdotal applications of medical therapy prior to attempted sperm retrieval for NOA [22]. Certainly, it is conceptually appropriate to treat low testosterone levels in men with NOA. However, retrospective data from a large series of men with NOA suggest that the benefits of treatment are quite limited, with men receiving medical therapy to raise testosterone levels having SRR of 51% (151/307) compared with men not receiving medical therapy prior to surgical intervention who had SRR of 61% (25/41) (*p* = 0.31) [56].

Medical therapy used to optimize testosterone levels aims to increase testosterone production and decrease estradiol levels. Elevated estradiol levels, typically greater than 60 pg/mL, can suppress hypothalamic gonadotropin secretion and subsequently inhibit testosterone production [6]. Testosterone-to-estradiol (T:E) ratios are normally greater than 10, and fertile men have a mean T:E ratio of approximately 15 (14.5 ± 1.2) [57,58]. Men with infertility have lower T:E ratios, with NOA men typically in the range of 7 (6.9 ± 0.6) and men with KS approximately 5 (4.4 ± 0.5) [58,59,60]. Gonadotropins can stimulate testosterone production and spermatogenesis in men with low gonadotropin levels secondary to congenital or acquired disorders [60]. hCG may be used as an luteinizing hormone (LH) substitute alone or in combination with an FSH analog (recombinant human FSH (rhFSH) or human menopausal gonadotropin (hMG)) to stimulate testis growth, testosterone production, and spermatogenesis [60]. AIs, including anastrozole and letrozole, prevent the actions of aromatase, which is present in peripheral tissues, in converting testosterone to estrogen. AIs have been shown to be effective medical therapy to increase SRR in men with KS and in infertile, non-KS men with abnormal T:E ratios [61,62]. SERMs, such as clomiphene citrate and tamoxifen, provide benefits by inhibiting the negative feedback exerted on the hypothalamic-pituitary-testis (HPT) axis by estrogen. With decreased negative inhibition, higher levels of LH and FSH can be achieved, resulting in increased serum testosterone levels and improved sperm production. Several studies have demonstrated positive impacts on semen parameters in infertile men taking clomiphene citrate [63,64]. 

Repair of clinical varicoceles has been demonstrated to improve serum testosterone levels, as well as spermatogenesis in men with oligozoospermia [48]. After varicocele repair in men with NOA, the potential improvement of spermatogenesis may result in enhanced SRR, although there is no high-level evidence to support such intervention [54].

## 5. Intracytoplasmic Sperm Injection for NOA

Prior to the advent of ICSI, men with NOA had no means for conceiving biological children. ICSI was first introduced in 1992, and three years later, in 1995, sperm retrieved from an NOA patient was used successfully with ICSI [65,66]. Since the development of ICSI, numerous studies have been performed to examine factors that may predict or be associated with increased ICSI success. 

### 5.1. Predictors of ICSI Outcomes

Limited pre-operative variables exist which predict success of SRR and ICSI. For predictors of SRR, patient age, serum hormone levels, and testicle size have been evaluated, however, conclusive evidence is lacking that any of these factors is predictive of successful sperm retrieval [67,68,69]. Testicular histopathology does provide some prognostic information for SRR, but it is not routinely recommended for diagnosis of NOA, as the diagnosis can be made clinically based on FSH > 7.6 and testis length < 4.5 cm in about 90% of men with this condition [22,68,70,71]. Similarly, no clinical or biochemical factors have been found to be predictive of ICSI outcomes [3]. Additionally, testis histology has not been shown to significantly influence clinical outcomes after ICSI [71]. There may, however, be an association of the number of sperm found at time of surgical retrieval with the number of clinical pregnancies [72]. Further work is needed to determine if any preoperative factors, in conjunction with female factors, can predict ICSI outcomes.

### 5.2. ICSI Outcomes in NOA Men

Understanding clinical outcomes after ICSI are important when counseling men with NOA and their partners prior to surgical sperm retrieval. A study derived from the National Assisted Reproductive Technology Surveillance System (NASS) found that men with infertility (including non-azoospermic and azoospermic men) had a clinical pregnancy rate (CPR) of 48% and live birth rate (LBR) of 40%, which was similar to rates in men with no infertility (CPR 44.9%, LBR 36.5%) [73]. A clear limitation of this study was that the various etiologies of male infertility were not specified or separately examined and thus, the CPR and LBR may not hold true for all etiologies of NOA. Although NOA men represent the most severe phenotype of those with male infertility, the majority of studies, similar to that previously described, have pooled men with NOA regardless of etiology which limits the overall generalizability of the data. A comprehensive summary of SRR, biochemical pregnancy rate (BCPR), CPR, and LBR from studies investigating ICSI outcomes between 1997 and 2020 in NOA men is presented in Table 3. 

A recent meta-analysis examining sperm retrieval as well as pregnancy and LBRs was performed [3]. This review compared SRR after conventional TESE (cTESE) with that after microTESE, and found that the per procedure SRR was 45–49%, and was not able to identify differences between conventional or microsurgical methods of sperm retrieval because the included studies did not include comparator trials [3]. Meta-regression analysis further demonstrated that SRR was independent of both age and hormonal parameters [3]. Testis volume greater than 12.5 mL was found to be associated with a greater than 60% chance of successful sperm retrieval with an accuracy of 86.2% [3]. The BCPR (diagnosed by positive serum hCG in the female partner) was 25–32% per ICSI cycle, and LBR was 20–28% [3]. It is important to note that the patient cohorts included in this meta-analysis were heterogenous with varying NOA etiologies, making the comparison of outcomes between cTESE and mTESE less valid. A previous meta-analysis which included fifteen comparative studies demonstrated a 17% higher likelihood of sperm retrieval success when performing mTESE compared to cTESE [2]. Additionally, it was noted that men who underwent mTESE had failed prior cTESE or TESA, which also may have underestimated the increase in sperm retrieval rate with mTESE [2]. Several, smaller studies have been performed examining NOA men based on underlying etiology, including KS, YCMD, malignancy, and cryptorchidism, and these will be discussed further.

#### 5.2.1. Klinefelter Syndrome

A meta-analysis of 37 studies found a cumulative SRR of 44% (39–48%) per TESE procedure in KS patients, with no significant difference between cTESE and mTESE [114]. ICSI outcomes were available for 29 of the 37 studies in the meta-analysis, and reported a cumulative CPR, defined by ultrasound detection of a gestational sac or heartbeat, of 43% (36–50%), and LBR of 43% (34–53%) per ICSI cycle [114]. SRR, CPR, and LBR in this analysis were independent of patient age at time of retrieval as well as testis volume, and serum hormone parameters [114]. Additionally, no differences between use of fresh versus frozen sperm were observed [114]. Again, it is important to note that this meta-analysis examined studies where the patient cohorts were not entirely made up of KS patients. In one of the largest published studies on SRR in KS patients, we report a SRR of 66% (Table 4) [61]. In our experience with KS patients, the appearance of tubules within the testis is unique amongst men with NOA. Instead of typically having sperm production throughout an individual seminiferous tubule, KS patients tend to have focal enlargement of otherwise sclerotic tubules within the testes. This appearance requires an intensive search within these typically very atrophic testes to find the millimeter-sized segments of tubules that may contain sperm. In addition, the number of sperm retrieved tends to be so small that sperm are typically not able to be frozen for later use. Therefore, the numbers of sperm obtained may be only adequate to inject available oocytes during a programmed, fresh in vitro fertilization (IVF) cycle. 

#### 5.2.2. Y-Chromosome Microdeletions

Little is known regarding ICSI and clinical outcomes in NOA men with YCMD given that many of these studies excluded men with YCMD or included men with YCMD in a larger cohort of azoospermic men (Table 3 and Table 4). SRRs differ drastically depending on the site of microdeletion. Sperm can be surgically retrieved in up to 70% of men with AZFc deletions and a subset may have low concentrations of sperm in the ejaculate, whereas no reports of sperm retrieval in men with complete AZFa or AZFb deletions have been effectively documented [17,116]. One study examining ICSI outcomes using ejaculated sperm demonstrated no significant difference in pregnancy, live birth, and miscarriage rates in men with AZFc microdeletions compared to those with other sources of infertility and no evidence of YCMD [117]. Another study found that men with AZFc microdeletions had a significantly increased fertilization rate when ejaculated sperm was used compared to testicular sperm [118]. With ejaculated sperm, pregnancy rate was 47% compared to 14% with testicular sperm [118]. Unfortunately, no predictors of successful sperm retrieval have been identified in this cohort, and it is important to inform couples that any male offspring will harbor the same Y-chromosome mutations [118,119].

#### 5.2.3. Chemotherapy-Associated NOA

In men treated with chemotherapy, SRR ranges from 37 to 60%, pregnancy rates 33–50%, and LBR 22–42% (Table 3 and Table 4) [79,92,100]. Additional studies have been performed examining SRRs and ICSI outcomes, but men treated for malignancy (either with chemotherapy or radiation) are often pooled with men who have NOA due to other underlying etiologies. Therefore, the reported clinical outcomes may not be accurate for men with azoospermia solely secondary to chemotherapy or radiation treatment. One retrospective study of male cancer survivors found that following chemotherapy or radiation treatment, approximately 57% were azoospermic [120]. This percentage is higher than expected in a full cohort of men treated with chemotherapy, as it reflects a referral bias to an infertility center for selected cancer survivors. The CPR was 38.6% and the LBR was 30.5% after ICSI [120]. Overall, SRR and ICSI outcomes are generally favorable for men who have undergone treatment for malignancy. In testis cancer survivors, 3% (who received chemotherapy) and 6% (who received radiation therapy) remained azoospermic two years after therapy [121]. With increasingly aggressive chemotherapy treatment regimens, the rates of persistent azoospermia are higher [122]. However, fertility preservation prior to cancer treatment is still highly recommended as it is minimally invasive for men and can potentially portend less invasive treatments for the female partner [22].

#### 5.2.4. Cryptorchidism

The mechanism underlying infertility in males with a history of cryptorchidism is not well understood. Men with bilateral cryptorchidism have a higher risk of infertility compared with those who have unilateral cryptorchidism [38,39,123]. Additionally, age at orchidopexy also affects sperm production and future fertility [40]. It is estimated that greater than 50% of men with a history of cryptorchidism will have varying degrees of spermatogenic failure and may require surgical sperm retrieval to conceive biological children [39,123]. 

Men who have had prior orchidopexy typically have substantial peri-testicular scar, no tunica vaginalis, and often have an abnormal lie or position of the testis with an anterior epididymis. Surgical treatment in these men also often reveals an atypical blood supply to the testis with variable patterns of vessels on the tunica albuginea. Within the testis, most tubules are typically sclerotic with distinctively different tubules containing isolated foci of spermatogenesis visible during microdissection. Studies examining ICSI outcomes solely in men with a history of cryptorchidism report SRR ranging from 52 to 85%, pregnancy rates ranging from 10–46%, LBR ranging from 33–100%, and miscarriage rates ranging from 6.3–8.1% (Table 2 and Table 3) [41,42,43,44,45]. Patients with cryptorchidism have a wide range of fertility potential and more studies of these individuals is needed to determine the mechanisms underlying their infertility so that patients can be counseled with accurate prognostic information. 

### 5.3. Sperm Effects on ICSI Outcomes

For men with severely impaired sperm production, embryo development appears to be adversely affected by the (testicular) source of sperm and level of sperm production with decreased development associated with higher FSH levels [124,125]. Men with NOA often have such low fertilization rates that a limited number of embryos exist on day 3. Therefore, transfer of day 3 embryos is routinely required, as further in vitro culture risks losing the embryos available for transfer on day 3. Of note, high-quality day 3 embryos have been suggested in some studies to be equivalent to day 5 blastocysts in terms of pregnancy and live birth rate [124]. However, existing data are limited with no interpretable data on embryo morphokinetic parameters, although some publications suggest that embryo development may be less efficient in me with lower sperm production [124,126]. Similarly, there are limited published data on blastocyst euploidy rates. Given the frequency of day 3 embryo transfer for couples where men have NOA, data obtained from the select group with blastocysts available for transfer may not reflect results that are generalizable for NOA patients as a whole. 

Advances in the understanding of sperm biology have revealed important paternal effects on embryo development and quality. Poor semen parameters have been demonstrated to negatively affect blastocyst formation rates after IVF and ICSI [124,126,127,128,129]. Ejaculated sperm has been found to produce higher fertilization and pregnancy rates than testicular sperm [130]. Additionally, when compared to men with obstructive azoospermia undergoing ICSI, men with NOA undergoing ICSI have lower rates of fertilization, blastocyst formation, implantation, and pregnancy [124,126,131,132]. 

## 6. Improving ICSI Outcomes from the Male Perspective

Optimization of hormone levels in NOA men may improve SRR [71]. Increased numbers of sperm may allow selection of more optimal sperm to be used for ICSI. Currently, various methods exist for improving sperm selection, including viability assays, cell sorting methods and enhanced microscopic analysis for selection of sperm for ICSI, although manual selection of individual sperm is the most common approach used in men with NOA [133]. 

Conventional sperm selection methods including the swim-up method, migration density, and density gradients rely heavily on the motility of sperm, and cannot be used for sperm retrieved from the testis as certain maturation processes have not occurred in testicular sperm and these sperm are immotile. Magnetic activated cell sorting (MACS) is a method of sperm selection using annexin V-conjugated magnetic beads to isolate viable sperm. A systematic review analyzing five prospective, randomized trials evaluating MACS compared to standard sperm selection methods (including swim-up and density gradient methods) found significantly increased pregnancy rates resulted after MACS [134]. Intracytoplasmic morphologically selected sperm injection (IMSI) is the process of selecting sperm at x6,600 magnification to examine motile sperm organellar morphology [133]. A prospective, randomized study examining ICSI and IMSI in couples with severe male factor infertility reported higher CPR with IMSI (39.2% IMSI vs. 26.5% ICSI, *p* = 0.004) [135]. This study along with other studies, including one meta-analysis, also demonstrated a decreased miscarriage rate and improved implantation rates with IMSI compared with ICSI [133,135]. However, a recent Cochrane review of the efficacy of IMSI compared with traditional ICSI did not provide any conclusive evidence to suggest that IMSI is superior to ICSI in terms of clinical outcomes (CPR, LBR) [136].

Additional work on novel techniques is underway that will further optimize the sperm chosen for oocyte injection. One promising technique is microfluidic sorting of sperm, which allows for analysis of sperm count, motility, and morphology on a microscopic level, allowing for the identification and selection of sperm with the best qualities [137,138]. Studies examining microfluidics-sorted sperm have demonstrated improved ICSI and clinical outcomes compared to sperm selected by conventional methods [138,139]. However, further work is needed to fully develop this technology for mainstream use [140]. Given the limited numbers of sperm available for retrieval from men with NOA, it is more challenging to apply sperm selection techniques that could be used for sperm samples from men with oligozoospermia.

## 7. Conclusions

ICSI has permitted NOA men, who were previously unable, to conceive biological children. Studies have demonstrated varying rates of clinical (pregnancy and live birth rates) outcomes likely due to a heterogenous population of men with NOA included in these studies, and future studies would benefit from etiology-specific outcome reporting. Understanding clinical outcomes after ICSI is important for prognostic information and counseling of these NOA men and their partners prior to undergoing invasive surgical procedures. Further work is needed to delineate the molecular mechanisms and genetic defects that underlie this severe reproductive phenotype.

## Figures and Tables

**Table 1:** Etiologies of non-obstructive azoospermia.

Etiology	Example
Idiopathic	
Genetic/Chromosomal	Klinefelter syndrome, Y-chromosome microdeletions
Iatrogenic/Surgical	Chemotherapy, Radiation therapy
Developmental/Structural	Cryptorchidism/Orchidopexy, Varicocele
Hormonal	Kallmann syndrome, hypogonadotropic hypogonadism, hyperprolactinemia/prolactinoma

**Table 2:** Medical therapy for hormonal optimization prior to sperm retrieval.

Class	Medication Name	Dose
AI	Anastrozole	1 mg/day
AI	Letrozole	2.5 mg/day
Gonadotropin	hCG	1500–3000 IU 2×/week
Gonadotropin	rhFSH	100–1500 IU 2–3×/week
SERM	Clomiphene Citrate	25–50 mg/day
SERM	Tamoxifen	20 mg/day

AI, aromatase inhibitor, SERM, selective estrogen receptor modulator; rhFSH, recombinant human follicle-stimulating hormone; hCG, human chorionic gonadotropin; IU, international unit.

**Table 3:** Studies reporting intracytoplasmic sperm injection outcomes in men with non-obstructive azoospermia.

Study	Year	NOA Etiology	Sperm Retrieval	SRR (%)	(BCPR (%)) CPR (%)	LBR (%)	MR (%)
Fahmy et al. [74]	1997	NR	cTESE	NR	(16.6) 19.2	NR	NR
Friedler et al. [75]	1997	NR	TESA TESE	43.0	29.0	NR	NR
Ben-Yosef et al. [76]	1999	NR	TESE *	60.0	21.7–27	13.0–25.0	6.7–8.7
Palermo et al. [77]	1999	NR	mTESE	63.9	49.1	NR	12.5
Mercan et al. [78]	2000	NR	TESA TESE	64.4	29–46	NR	20.7–24.2
Chan et al. [79]	2001	Chemotherapy	cTESE mTESE	45.0	(44.5) 33.3	22.2	NR
Damani et al. [80]	2002	Chemotherapy	cTESE	65.2	60.0	53.0	NR
Friedler et al. [81]	2002	NR	cTESE	39.0–85.0	16.0–19.0	67.0–80.0	NR
Mátyás et al. [82]	2002	NR	cTESE	69.6	26.7	NR	NR
Bailly et al. [83]	2003	NR	cTESE	35.0	18.0	81.8	9.0
Mansour et al. [84]	2003	NR	cTESE	56.1	13.6–24.1	NR	NR
Meseguer et al. [85]	2003	Chemotherapy	cTESE	41.7	20.0	20.0	NR
Osmanagaoglu et al. [86]	2003	NR	TESE *	NR	NR	13.9	NR
Raman et al.—a [42]	2003	Cryptorchidism	cTESE mTESE	74.0	46.0	43.0	NR
Raman et al.—b [42]	2003	NR	cTESE mTESE	58.0	44.0	36.0	8.1
Vernaeve et al.—a [43]	2004	NOA (excluded cryptorchidism)	cTESE	33.3%	(20.7) 10.9	10.9	NR
Vernaeve et al.—b [43]	2004	Cryptorchidism	cTESE	51.9	(28.1) 17.2	17.2	NR
Aydos et al. [87]	2005	Cryptorchidism, idiopathic, nontestis cancer, RT, trauma, mumps, orchitis, chromosome anomaly	mTESE	57.0	36.0	NR	NR
Giorgetti et al. [88]	2005	NR	cTESE	46.0	35.3	25.0–29.0	NR
Mitchell et al. [89]	2005	NR	cTESE	N/A	8.7–26.7	17.4–33.3	NR
Wu et al. [90]	2005	NR	cTESE	76.7	33.3–62.5	33.3–41.7	0–20.8
Everaert et al. [91]	2006	NR	MESA mTESE	35.4	(13.2) 9.4	7.5	NR
Hibi et al. [92]	2007	Chemotherapy	mTESE	60.0	NR	40.0	NR
Mitchell et al. [93]	2007	NR	cTESE	N/A	26.0	13.3	NR
Kanto et al. [94]	2009	NR	mTESE	42.5	52.9	NR	NR
Ravizzini et al. [95]	2008	NR	mTESE	57.1	(50.0) 40.0	40.0	NR
Ishikawa et al. [96]	2009	NR	mTESE	N/A	(36.8) 30.9	26.5	NR
Wiser et al. [44]	2009	Cryptorchidism	cTESE	59.5	30.8–41.2	75.0–80.0	NR
Yarali et al.—a [97]	2009	non-KS	mTESE	44.0	(41.0) 33.0	26.0	NR
Yarali et al.—b [97]	2009	KS	mTESE	56.0	(61.0) 39.0	28.0	NR
Boitrelle et al. [98]	2011	Cryptorchidism, KS, YCMD, Y inversion, malignancy, idiopathic chemotherapy/RT	cTESE	53.2	42.7	37.0	7.9 5.3 ^€^
Hauser et al. [99]	2011	NOA + cryptozoospermia	cTESE	N/A	(19.1–42.9) 12.8–42.9	12.8–42.9	NR
Hsiao et al. [100]	2011	Chemotherapy	mTESE	37.0	50.0	42.0	NR
Ashraf et al. [101]	2013	NR	mTESE	50.0	40.0	NR	NR
Choi et al.—a [102]	2013	NOA + AZFc YCMD	cTESE	21.0	NR	19.5	NR
Choi et al.—b [102]	2013	AZFc YCMD	cTESE	26.6	NR	24.3	NR
Karacan et al. [103]	2013	NR	mTESE	54.9	31.3	28.9	7.6
Arafa et al. [104]	2014	Familial and non-familial idiopathic NOA	mTESE	37.4	13.9	NR	NR
Esteves et al. [105]	2014	NR	mTESE	41.4	27.8	19.9	28.6
Karacan et al. [106]	2014	NR	mTESE	48.9	16.6–30.7	16.6–28.2	8.3
Aydin et al. [107]	2015	NR	mTESE	58.6	44.6	NR	NR
Tsai et al. [45]	2015	Cryptorchidism	TESE *	N/A	45.6	32.9	6.3
Vloeberghs et al. [108]	2015	NR	cTESE	40.5	(27.7–34) 21.7–26.7	20.6–25.3	NR
Ko et al. [109]	2016	NR	cTESE mTESE	44.9	(37.5) 30	25.0	NR
Alfano et al. [110]	2017	Idiopathic NOA	mTESE	48.9	21.7	13.0	NR
Arafa et al.—a [111]	2018	Idiopathic NOA + AZFc YCMD	TESE *	63.2	25.7	NR	NR
Arafa et al.—b [111]	2018	Idiopathic NOA	TESE *	65.8	26.6	NR	NR
Yu et al. [112]	2018	NR	mTESE	38.4	(34.3) 49.1	24.6	20.7
Chen et al. [41]	2019	Idiopathic, KS, YCMD, cryptorchidism, mumps orchitis, chemotherapy	mTESE	40.3	51.0–55.8	NR	NR
Yamaguchi et al.—a [113]	2020	NOA (excluded AZFc YCMD)	mTESE	74.0	28.9	NR	20.2
Yamaguchi et al.—b [113]	2020	AZFc YCMD	mTESE	20.4	24.7	NR	26.3

AZFRc, azoospermia factor region deletion in locus c; SRR, sperm retrieval rate; BCPR, biochemical pregnancy rate (elevated serum hCG); CPR, clinical pregnancy rate (heartbeat or gestational sac detectable by ultrasound); LBR, live birth rate; MR, miscarriage rate; NOA, non-obstructive azoospermia; NR, not reported; TESA, testicular sperm aspiration; cTESE, conventional TESE; mTESE, microdissection TESE; TESE *—type of TESE not specified; ^€^, ectopic pregnancy rate; KS, Klinefelter syndrome; YCMD, Y-chromosome microdeletion; RT, radiation therapy; MESA, microsurgical epiddiymal sperm aspiration. “a” and “b” were used to denote different patient cohorts examined within one study.

**Table 4:** Sperm retrieval rates in non-obstructive azoospermia by etiology.

NOA Etiology	Weill Cornell Medicine (P.N.S.)	Other Reports
Idiopathic	48.5% [46]	37.4–65.8% [104,111]
Klinefelter syndrome	61–66% [46,61]	44% [114]
YCMD (AZFc)	67∓75% [17,46]	20.4–54.8% [102,113,115]
Chemotherapy	42% [46]	37–60% [79,92,100]
Cryptorchidism	62% [46]	52–85% [41,42,43,44,45]
Overall	48% [46]	45–49% [3]

P.N.S., Peter N. Schlegel, attending urologist at Weill Cornell Medicine.

## Data Availability

Not applicable.
